# Does the Current Evidence for Lecanemab Mechanism Support a Rationale for Continued Lecanemab Dosing?

**DOI:** 10.1002/alz.092090

**Published:** 2025-01-09

**Authors:** Dennis J. Selkoe, Shobha Dhadda, Michael C. Irizarry, Lynn D Kramer

**Affiliations:** ^1^ Ann Romney Center for Neurologic Diseases, Department of Neurology, Brigham and Women’s Hospital, Harvard Medical School, Boston, MA USA; ^2^ Eisai Inc., Nutley, NJ USA

## Abstract

Alzheimer’s disease pathophysiology is believed to involve various abnormalities, including those of amyloid beta (Ab) peptide and tau processing, inflammation, oxidative stress, and vascular risk factors. Aβ peptides exist in a dynamic continuum of conformational states from monomeric Aβ, to soluble progressively larger Aβ assemblies that include a range of low molecular weight oligomers to higher molecular weight protofibrils, and finally to insoluble fibrils (plaques). Various lines of evidence support the “amyloid hypothesis” that Aβ plays a central role in the pathogenesis of AD, and several immunotherapies have been developed to interact with this cascade in various different places which may reduce the number of soluble aggregates and insoluble Aβ fibrils deposited in the brain. Lecanemab is a novel humanized immunoglobulin G1 (IgG1) anti‐amyloid monoclonal antibody with highest affinity for Aβ protofibrils, a particularly toxic Aβ species. Lecanemab distinguishes itself from other anti‐amyloid antibodies in that it selectively targets large soluble protofibrils relative to monomers (greater than 1000‐fold over Aβ monomers), with preferential activity over insoluble fibrils (up to 10‐fold over fibrils). In the phase 3 Clarity AD study, lecanemab demonstrated a consistent slowing of decline over 18 months in clinical (global, cognitive, functional, and quality of life) outcomes which if used early in the clinical paradigm has shown to slow disease progression for up to 30 months, and reduction in brain amyloid in early Alzheimer’s disease. Herein, we will highlight the mechanism‐based rationale for continued lecanemab dosing, providing both the current supportive evidence and outstanding questions. Relevant Alzheimer’s disease background will be discussed, including the impact of the latest diagnostic criteria. We will share insights on Alzheimer’s disease pathophysiology, including how the ongoing/chronic nature of the disease requires continuous therapy. In addition, we will review the latest data on lecanemab mechanism and overview of the mechanistic rationale for development of lecanemab Aβ immunotherapy. The role of tau aggregates, a predictive biomarker for the emergence of neurodegeneration, will be explored. Additional insights on continued dosing based on current knowledge of lecanemab mechanism will be shared. An overview of the mechanistic differences and their implications among anti‐amyloid antibodies will be presented.

